# Multicenter study of re‐irradiation using carbon‐ions for head and neck malignancies after photon radiotherapy

**DOI:** 10.1002/cam4.4741

**Published:** 2022-04-07

**Authors:** Daiki Takahashi, Yusuke Demizu, Masashi Koto, Nobuteru Kubo, Hiroaki Suefuji, Hiroaki Ikawa, Tatsuya Ohno, Yoshiyuki Shioyama, Tomoaki Okimoto, Hiroshi Tsuji, the Japan Carbon‐Ion Radiation Oncology Study Group

**Affiliations:** ^1^ Department of Radiology Hyogo Ion Beam Medical Center Tatsuno Japan; ^2^ Department of Radiation Oncology Hyogo Ion Beam Medical Center Kobe Proton Center Kobe Japan; ^3^ QST Hospital National Institutes for Quantum Sciences and Technology Chiba Japan; ^4^ Gunma University Heavy Ion Medical Center Maebashi Japan; ^5^ Ion Beam Therapy Center SAGA‐HIMAT Foundation Tosu Japan

**Keywords:** clinical cancer research, head and neck, multicenter study, salvage treatment

## Abstract

**Purpose:**

The goal of this multicenter retrospective study of patients with head and neck malignancies was to evaluate the efficacy and safety of carbon‐ion (C‐ion) radiotherapy (RT) after photon RT.

**Methods:**

We enrolled 56 patients with head and neck malignancies who underwent re‐irradiation (re‐RT) using C‐ions between November 2003 and March 2019, treated previously with photon RT. The tumors at re‐RT were located in the sinonasal cavities (*n* = 20, 35.7%), skull base (*n* = 12, 21.4%), and orbit (*n* = 7, 12.5%). The tumors at the initial RT were located in the sinonasal cavities (*n* = 13, 23.2%), skull base (*n* = 9, 16.1%), and orbit (*n* = 9, 16.1%). The median period between the initial RT and re‐RT was 41 (4–568) months. The most common histology of re‐RT was squamous cell carcinoma (*n* = 11, 19.6%). The most commonly used protocol was 57.6 Gy (relative biological effectiveness) in 16 fractions (*n* = 23, 41.1%). Surgery preceded re‐RT in three patients (5.4%). One patient with malignant melanoma received concurrent chemotherapy.

**Results:**

The 2‐year local control, progression‐free survival, and overall survival rates were 66.5%, 36.9%, and 67.9%, respectively. The median follow‐up time was 28 months. Two patients (3.6%) developed grade ≥ 3 acute toxicities, and 14 (25.0%) developed grade ≥ 3 late toxicities. A single patient had confirmed grade 5 dermatitis with infection.

**Conclusion:**

Re‐RT using C‐ions for head and neck malignancies after photon RT is an effective treatment with tolerable toxicity.

## Graphical abstract

The 2‐year local control, progression‐free survival, and overall survival rates were 66.5%, 36.9%, and 67.9%, respectively. Two patients (3.6%) developed grade ≥ 3 acute toxicities, and 14 (25.0%) developed grade ≥ 3 late toxicities.

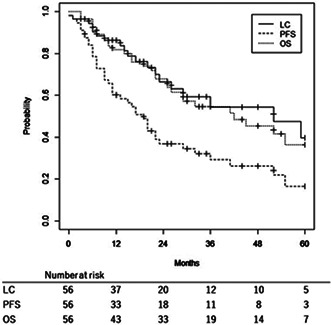

## 1 INTRODUCTION

Treatment strategies for head and neck malignancies must consider functional and cosmetic preservation, as well as tumor control. Photon radiotherapy (RT), widely used for functional and cosmetic preservation, can be added to the standard for treatment protocol for unresectable tumors^1^ and postoperative adjuvant therapies.^2^ However, the management of malignancies in patients with prior photon RT is challenging. Several advanced treatments (including stereotactic RT and intensity‐modulated RT) have been developed and re‐irradiation (re‐RT) for head and neck malignancies has improved.^3^, ^4^

High energy X‐rays, used in conventional RT, have the ability to penetrate the human body, while carbon ions (C‐ions) can reach desirable depths.^5^ Additionally, C‐ions present a Bragg peak, the peak region that occurs where their range ends and have little dose distribution beyond their designated depth. C‐ions have the ability to achieve dose localization for deep‐seated tumors, being accelerated by the designed energy to develop a high dose at their target depth.

C‐ions are classified as high‐linear energy transfer (LET) radiation and exhibit higher relative biological effectiveness (RBE) than X‐rays.^5^ Because plateaus of C‐ions in the superficial layer of a body show low LET and RBE, C‐ion RT (CIRT) can achieve both high dose radiation at the target region and high biological effects.^5^ CIRT is, therefore, considered useful for photon‐resistant tumors.^6^, ^7^

Based on these characteristics, re‐RT using C‐ions for head and neck malignancies can be considered to have efficacy against photon‐resistant tumors and superiority over photon RT in terms of safety. In a multicentric in silico trial of re‐RT for head and neck cancers, it was reported that C‐ions showed better dose localization than protons and X‐rays.^8^

Single institutional outcomes of re‐RT using C‐ions for head and neck malignancies have been reported in Germany, China, and Japan. Held et al., in a clinical study of patients previously treated with a course of irradiation including CIRT, reported that the median overall survival (OS) was 26.1 months, and 14.5% of patients experienced grade ≥3 late toxicity.^9^ Gao et al. reported results in patients previously treated with definitive photon‐based RT and showed that the 1‐year OS rate was 95.9%.^10^ Hayashi et al. reported clinical results in patients previously treated with CIRT, and showed that the 2‐year OS rate was 59.6%; 37.5% of patients experienced grade ≥3 late toxicities.^11^

In Japan, several CIRT centers, including the Hyogo Ion Beam Medical Center, QST Hospital, SAGA‐HIMAT Foundation, and Gunma University Heavy Ion Medical Center, treat head and neck tumors. We conducted a multicentric study to assess retrospectively clinical data of CIRT for head and neck malignancies after photon RT (J‐CROS study: 1903 HN).

## 2 METHODS

### 2.1 Eligibility

Patients provided informed consent for the use of personal data. This study was approved by each of the relevant institutional review boards and was conducted in compliance with the Declaration of Helsinki. The identification number of this trial is UMIN000038950. Fifty‐six patients were enrolled, each had undergone re‐RT to the head and neck using C‐ions between November 2003 and March 2019, treated previously with photon RT.

### 2.2 Carbon‐ion radiotherapy

The gross tumor volume (GTV) was based on computed tomography, positron emission tomography, and magnetic resonance imaging findings. The clinical target volume (CTV) was set as the GTV plus 0–5‐mm margins. The planning target volume (PTV) had 2–5‐mm margins around the CTV.

The doses of C‐ions were shown as a photon‐equivalent dose in Gy (RBE), which was defined as the physical dose multiplied by the RBE of the C‐ion.^12^

### 2.3 Clinical outcome and toxicity

We determined local control (LC) if the PTV showed no tumor regrowth. If neither local recurrence nor metastasis in the regional lymph nodes was observed, locoregional control was determined. Toxicities were evaluated based on the Common Terminology Criteria for Adverse Events, version 4.0, and data on grade ≥3 acute toxicity and grade ≥2 late toxicities were collected. All patients underwent restaging based on the eighth edition of the tumor‐node‐metastasis staging system (International Union Against Cancer, 2017).

### 2.4 Statistical analyses

The LC, locoregional control, progression‐free survival (PFS), and OS rates were computed from the first day of CIRT and analyzed using the Kaplan Meier method. All statistical tests were two‐sided. Univariate analyses, using the log‐rank test, were conducted to investigate prognostic factors for LC, PFS, and OS. All factors with statistically significant associations in the log‐rank test were analyzed by the multivariate analysis using the Cox‐proportional hazards model. Statistical significance was set at *p* < 0.05. The number of patients with grade ≥3 late toxicities was counted to determine the cumulative incidence using the Kaplan–Meier method. Statistical analyses were completed using R 4.0.3 (R Core Team).

## 3 RESULTS

### 3.1 Cohort

Table 1 shows summaries of the patients’ characteristics. The tumors treated with re‐RT using C‐ions were located in the sinonasal cavities (*n* = 20, 35.7%), skull base (*n* = 12, 21.4%), and orbit (*n* = 7, 12.5%). The tumors treated with the initial RT were located in the sinonasal cavities (*n* = 13, 23.2%), skull base (*n* = 9, 16.1%), and orbit (*n* = 9, 16.1%). The median period between initial RT and re‐RT was 41 (4–568) months. Twenty‐four patients (42.9%) were treated for <3 years from the initial RT. PTV overlap was confirmed in 48 patients (85.7%). The most common histology of the re‐RT was squamous cell carcinoma (*n* = 11, 19.6%). Thirteen (23.2%) patients presented with a second primary tumor, while 43 (76.8%) presented with recurrence of the original tumors. The most commonly used radiation protocol was 57.6 Gy (RBE) in 16 fractions (*n* = 23, 41.1%). Surgery preceded re‐RT in three patients (5.4%). Six patients received neoadjuvant chemotherapy, and these regimens included TS‐1 + docetaxel (DTX), cisplatin (CDDP) + fluorouracil (5‐FU), dacarbazine + nimustine + vincristine (DAV), nedaplatin (NDP) + 5‐FU, cetuximab + CDDP + 5‐FU, and unknown. One patient received concurrent chemotherapy with DAV.

**TABLE 1:** Patients' characteristics

Factors	Patients
No. of patients	56 (100.0)
Sex, *n* (%)	
Male	33 (58.9)
Female	23 (41.1)
Age, y (%)	
Median	62
Range	17–82
<60	25 (44.6)
≧60	31 (55.4)
Performance status, *n* (%)	
0	25 (44.6)
1	26 (46.4)
2	4 (7.1)
3	0 (0.0)
4	1 (1.8)
Site of irradiation (Re‐RT), *n* (%)	
Sinonasal cavities	20 (35.7)
Skull base	12 (21.4)
Orbit	7 (12.5)
Major salivary gland	3 (5.4)
Acoustic organ	3 (5.4)
Pharynx	2 (3.6)
Oral cavity	2 (3.6)
Others	7 (12.5)
Site of irradiation (Initial RT), *n* (%)	
Sinonasal cavities	13 (23.2)
Skull base	9 (16.1)
Orbit	9 (16.1)
Pharynx	7 (12.5)
Oral cavity	5 (8.9)
Major salivary gland	3 (5.4)
Acoustic organ	2 (3.6)
Others	8 (14.3)
Tumor classification, *n* (%)	
T0	2 (3.6)
T1	10 (17.9)
T2	5 (8.9)
T3	2 (3.6)
T4	32 (57.1)
Unclassified	5 (8.9)
Node classification, *n* (%)	
N0	48 (85.7)
N1	2 (3.6)
N2	0 (0.0)
N3	1 (1.8)
Unclassified	5 (8.9)
Interval between initial RT and re‐RT (m)	
Median	41
Range	4–568
<36	24 (42.9)
≧36	32 (57.1)
Type of radiation (Initial RT), *n* (%)	
X‐ray	47 (83.9)
Gamma ray	9 (16.1)
GTV (cm^3^)	
Median	27.1
Range	3.6–219.0
<27	28 (50.0)
≧27	28 (50.0)
PTV (cm^3^)	
Median	88.8
Range	15.0–424.0
<89	28 (50.0)
≧89	28 (50.0)
Low risk PTV *n* (%), (cm^3^)	
Yes	8 (14.3)
Median	175.0
Range	24.0–533.0
No	48 (85.7)
PTV overlap	
Yes	48 (85.7)
No	2 (3.6)
Unknown	6 (10.7)
Histology (Re‐RT), *n* (%)	
Squamous cell carcinoma	11 (19.6)
Adenoid cystic carcinoma	8 (14.3)
Chordoma	6 (10.7)
Malignant melanoma	5 (8.9)
Mucoepidermoid carcinoma	5 (8.9)
Adenocarcinoma	3 (5.4)
Rhabdomyosarcoma	3 (5.4)
Others	15 (26.8)
Histology (Initial RT), *n* (%)	
Squamous cell carcinoma	13 (23.2)
Adenoid cystic carcinoma	8 (14.3)
Chordoma	6 (10.7)
Malignant melanoma	4 (7.1)
Adenocarcinoma	3 (5.4)
Retinoblastoma	3 (5.4)
Others	19 (33.9)
Second primary tumors, *n* (%)	
Yes	13 (23.2)
No	43 (76.8)
Dose fractionation (Gy [RBE]/number of fractions)	
57.6/16	23 (41.1)
60.8/16	7 (12.5)
64/16	5 (8.9)
70.4/16	4 (7.1)
60/30	4 (7.1)
57.6/12	3 (5.4)
Others	10 (17.9)
Irradiation system	
Active scanning	12 (21.4)
Passive scattering	44 (78.6)
Surgery prior to re‐RT, *n* (%)	
Yes	3 (5.4)
No	53 (94.6)
Chemotherapy, *n* (%)	
Neo‐adjuvant	6 (10.7)
Concurrent	1 (1.8)
No	49 (87.5)

Abbreviations: GTV, Gross tumor volume; PTV, Planning target volume; RBE, relative biological effectiveness; Re‐RT, re‐irradiation; RT, radiotherapy.

### 3.2 Clinical outcomes

**FIGURE 1:**
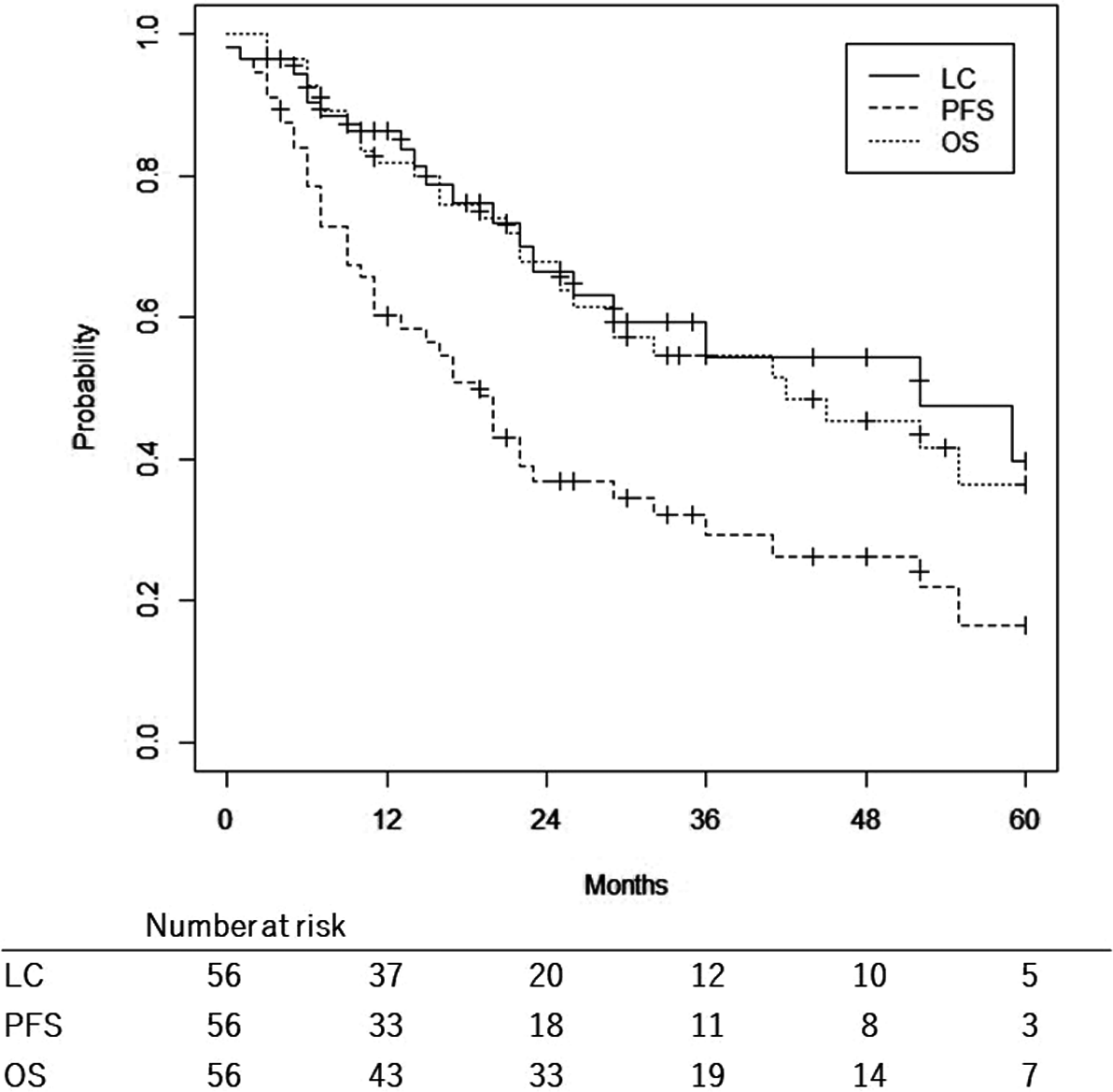
Kaplan–Meier curves of local control, progression‐free survival, and overall survival following re‐irradiation using carbon ions for head and neck malignancies. LC, local control; PFS, progression‐free survival; OS, overall survival

The median follow‐up time was 28 (3–147) months. The 2‐year LC, PFS, and OS rates were 66.5%, 36.9%, and 67.9%, respectively (Figure 1). The 2‐year locoregional control rate was 52.3%. The 2‐year cumulative incidence of distant metastasis was 31.0%. In patients with recurrent tumors, the 2‐year LC, PFS, and OS rates were 62.0%, 35.2%, and 70.9%, respectively. In patients with second primary tumors, the 2‐year LC, PFS, and OS rates were 84.6%, 42.3%, and 55.9%, respectively. No significant difference was observed between recurrent tumors and second primary tumors (Figure S1).

Table 2 shows the result of the analyses for each factor. The univariate analyses revealed that surgery prior to re‐RT and the interval between initial RT and re‐RT (< 36 months) were prognostic factors of a low LC. Additionally, the site of irradiation at initial RT (sinonasal cavities) and the interval between the initial RT and re‐RT (<36 months), were prognostic factors of a low PFS and OS. In the multivariate analysis based on the Cox proportional hazards model, all aforementioned factors were identified as significant predictors of LC, PFS, and OS.

**TABLE 2:** Univariate and multivariate analysis for OS, PFS, and LC

Factor	Univariate				Multivariate		
	*p* value				*p* value		
	*n*	OS	PFS	LC	OS	PFS	LC
Sex (y)							
Male	33	0.1	0.9	0.5			
Female	23						
Age							
<60	25	0.4	0.8	1			
≧60	31						
Performance status							
1	26	0.1	0.8	0.7			
Others	30						
Site of irradiation (Re‐RT)							
Sinonasal cavities	20	0.7	0.2	0.2			
Others	36						
Site of irradiation (Initial RT)							
Sinonasal cavities	13	0.001	0.006	0.4	0.015	0.048	
Others	43						
Tumor classification							
T4	32	0.3	0.3	0.8			
Others	24						
Node classification							
N0	48	0.9	0.9	0.4			
Others	8						
Interval between initial RT and re‐RT (m)							
<36	24	0.007	0.0003	0.001	0.044	0.0021	0.0028
≧36	32						
Type of radiation (Initial RT), *n* (%)							
X‐ray	47	0.5	0.4	0.4			
Others	9						
GTV (cm^3^)							
<27	28	0.08	0.07	0.1			
≧27	28						
PTV (cm^3^)							
<89	28	0.2	0.8	0.2			
≧89	28						
Low risk PTV							
Yes	8	0.7	0.5	0.3			
No	48						
Second primary tumors							
Yes	13	0.7	0.7	0.5			
No	43						
Dose fractionation (Gy [RBE]/number of fractions)							
57.6/16	23	0.9	0.6	1			
Others	33						
Irradiation system							
Active scanning	12	0.9	1	0.8			
Passive scattering	44						
Surgery prior to re‐RT							
Yes	3	0.7	0.2	0.02			0.031
No	53						
Chemotherapy							
Yes	7	0.2	0.2	0.9			
No	49						
Late toxicity (Grade ≧ 3)							
Yes	14	0.8	1	0.9			
No	42						

Abbreviations: GTV, gross tumor volume; LC, local control; OS, overall survival; PFS, progression‐free survival; PTV, planning target volume; RBE, relative biological effectiveness; Re‐RT, re‐irradiation; RT, radiotherapy.

### 3.3 Acute and late toxicities

Two patients (3.6%) developed grade ≥3 acute toxicities: one grade 3 acute dermatitis, and the other grade 3 acute pharyngeal mucositis. All 56 patients completed re‐RT using C‐ions.

**FIGURE 2:**
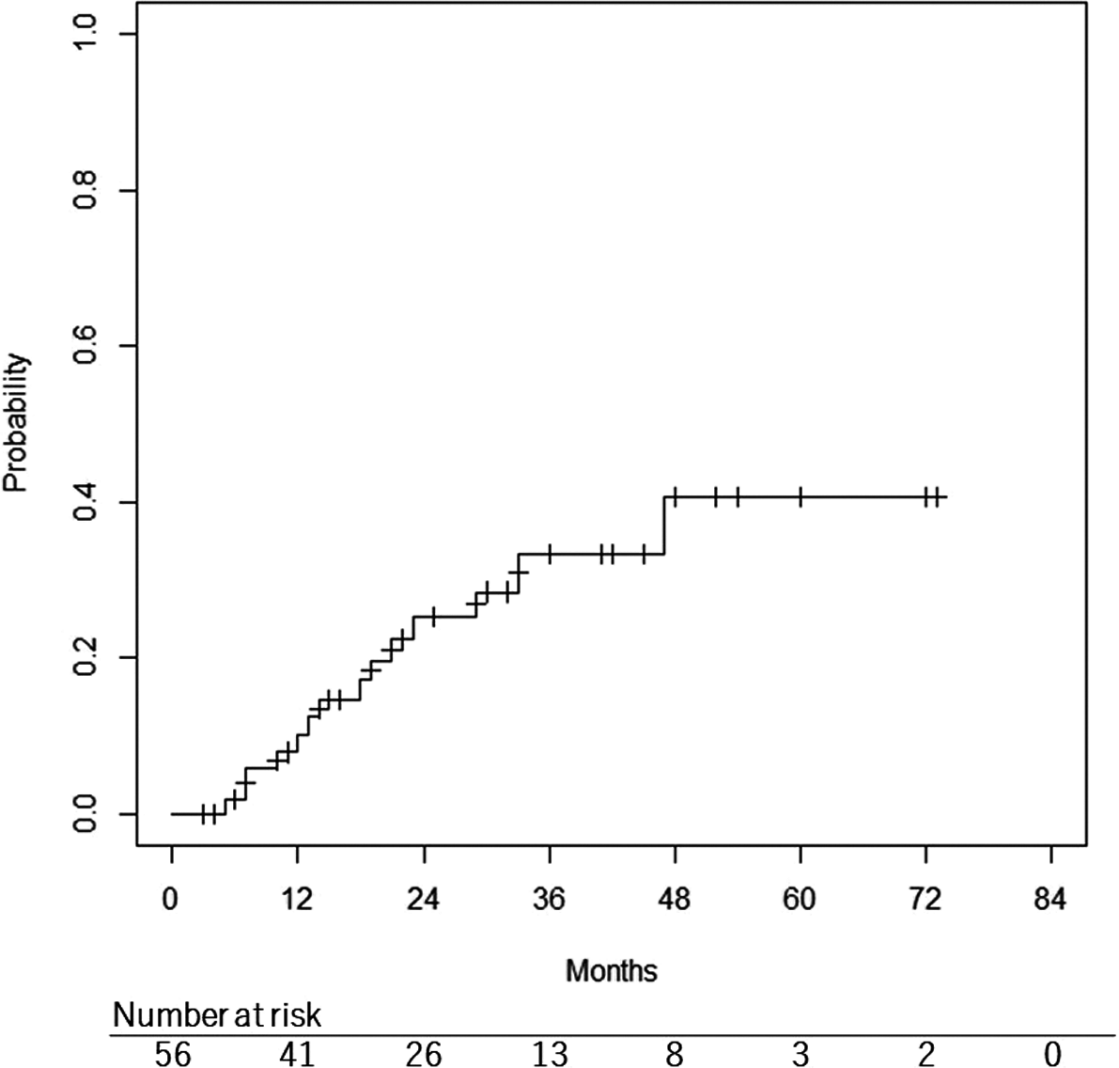
Cumulative incidence of grade ≥3 late toxicities with the Kaplan–Meier method

Regarding late toxicities, the 2‐year cumulative incidence of grade ≥3 late toxicities was 25.2% using the Kaplan Meier method. (Figure 2). Fourteen patients (25.0%) developed grade ≥3 toxicities. Grade 5 dermatitis with infection was confirmed in one patient. Grade 4 vision loss, hemorrhage, and mucositis developed in four, one, and one patient(s), respectively. Vision loss in three patients developed only on the affected side. All late toxicities with their details are summarized in Tables S1–S3.

## 4 DISCUSSION

Re‐RT is challenging because of the complexity involved in the tolerance of various normal tissues. The decision between achieving therapeutic efficacy and minimizing toxicities must be carefully considered. Proton RT and CIRT are expected to improve dose conformity,^13^ and further studies are required to evaluate the benefit of re‐RT. This may be the first multi‐institutional study to examine the clinical outcomes and toxicities of re‐RT using C‐ions for head and neck malignancies. It has been reported that re‐RT for head and neck malignancies is tolerable and feasible.^9^, ^10^, ^14^, ^15^, ^16^, ^17^, ^18^, ^19^ We evaluated the efficacy and toxicities in our cohort, and our findings demonstrated that re‐RT using C‐ions may represent an effective and tolerable treatment (Table 3).

**TABLE 3:** Comparison of our findings with those of historical studies

Study	Modality	Study design	*n*	Median follow up periods (month)	Treatment methods (%)	SCC (%)	OS (%): year	LC (%: year)	Proportion of patients with severe late toxicities (%)
Our study	Carbon	M	56	28	RT (98.2) and CCRT (1.8)	19.6	67.9: 2 y	66.5: 2 y	25.0: Grade ≥ 3
Held et al.^9^	Carbon	S	229	28.5	RT	26.2	59.2: 1.5 y	44.7: 1.5 y	14.5: Grade ≥ 3
Held et al.^14^	Carbon	S	32	18.1	RT	0	77.4: 1 y	66: 1 y	0: Grade ≥ 3
Gao et al.^10^	Carbon	S	141	14.7	RT and CCRT	75.3	95.9: 1 y	84.9: 1 y	approximately 10
Spencer et al.^15^	Photon	M	79	—	CCRT	77.2	15.2: 2 y	—	—
Ward et al.^16^	Photon (IMRT)	M	412	10.4	RT (25) and CCRT (75)	—	40.0: 2 y	—	—
Romesser et al.^17^	Proton	M	92	10.4	RT (52.2) and CCRT (47.8)	56.5	65.2: 1 y	—	7.2: Grade 4 2.9: Grade 5
Phan et al.^18^	Proton	S	60	13.6	RT (26.7) and CCRT (73.3)	66.7	69.0: 2 y	—	20.0: Grade 3

Abbreviations: CCRT, concurrent chemoradiotherapy; IMRT, intensity‐modulated radiotherapy; LC, local control; M, multi‐institution; *n*, number of patients; OS, overall survival; PFS, progression‐free survival; RBE, relative biological effectiveness; RT, radiotherapy; S, single‐institution; SCC, squamous cell carcinoma.

The most limiting factor of re‐RT is grade 5 toxicities. McDonald et al. reported that the probability of carotid rupture was 2.6%, and 76% of them were grade 5^20^ in patients receiving re‐RT using photons. Grade 5 carotid ruptures were observed in both acute and late toxicities. Gao et al. reported that grade 5 mucosal necrosis led to hemorrhage in four patients receiving re‐RT using C‐ions as late toxicities.^10^ In this study, one patient developed grade 5 dermatitis with infection as a late toxicity. As previously reported, severe skin necrosis led to death due to sepsis.^21^

Grade 4 vision loss, hemorrhage, and mucositis developed in four, one and one patient(s), respectively. Vision loss can develop from the use of any type of beam.^22^, ^23^, ^24^ Even with CIRT, it is sometimes difficult to avoid vision loss if an optic nerve is adjacent to a tumor.^22^, ^23^ All patients who had the potential to develop vision loss were informed about the risks of vision loss induced by re‐RT.

Acute toxicity is also the most serious side‐effect in RT. Held et al. reported that treatment was canceled due to acute laryngeal edema in patients receiving re‐RT using C‐ions.^9^ It has been reported that the completion rates of re‐RT for recurrent head and neck cancer cases using photons, protons, and C‐ions were 95%,^16^ 94.6%^17^ to 98.3%,^18^ and 96.9%,^14^ respectively. In this study, all 56 patients completed re‐RT using C‐ions.

Comparing the toxicities and effectiveness found in this study with those of previous studies is complicated because of this study's longer follow‐up time (median follow‐up, 28 months). As indicated in Table 3, there was one study with a comparable follow‐up period reported by Held et al. (median follow‐up, 28.5 months).^9^ Our study showed that the 2‐year LC and OS rates on re‐RT using C‐ions were 66.5% and 67.9%, respectively. These clinical outcomes appear to be better than those reported by Held et al. (1.5‐year LC, 44.7% and OS, 59.2%) but lead to a higher grade ≥3 late toxicity (25.0% vs. 14.5%). There is no clear solution to prioritize minimizing toxicities or achieving efficacy, and the treatment plan must be decided with consideration for an individual patient's condition.

Photon RT plus concurrent cisplatin treatment is the standard for head and neck squamous cell carcinoma.^2^ Concurrent chemotherapy was administered with re‐RT using photons and protons.^15^, ^16^, ^17^, ^18^ However, there are only a few reports on concomitant chemotherapy with CIRT.^25^, ^26^, ^27^, ^28^ In this study, only one patient with malignant melanoma received concurrent treatment with dacarbazine, nimustine, and vincristine (DAV therapy), as previously reported.^27^ Our clinical outcomes were not clearly inferior to outcomes of re‐RT using protons in combination with concurrent chemotherapy.^17^, ^18^ Gao et al. published the 1‐year results of re‐RT using C‐ions, including a cohort with concurrent chemotherapy, and long‐term data are expected.^10^

Proton beams have similar physical properties to those of CIRT and may be useful for re‐irradiation.^17^, ^18^ However, the rapid distal fall‐off and sharper lateral penumbra of CIRT can achieve more conformal irradiation than that in proton therapy.^5^, ^8^ Therefore, CIRT is considered more advantageous for malignancies located close to organs at risk.

There was no significant difference in LC, PFS, and OS between patients with recurrent tumors and those with second primary tumors. Our results indicate that a longer RT interval significantly improved OS, PFS, and LC, a finding also informed by Ward et al.^16^ and Held et al.^9^, ^14^ Patients who underwent surgery prior to re‐RT had a significantly lower LC rate, but there were only a few such patients. The site of the initial RT (sinonasal cavities) was identified as a significant prognostic factor of worse PFS and OS. Because this factor did not significantly affect LC, it is possible that this significant difference was not correlated with CIRT. Further studies are required to understand these two factors.

Our study has two limitations: our data were retrospectively analyzed and various radiation dosages were adopted, which may have influenced the clinical outcomes.

## 5 CONCLUSIONS

Our findings suggest that re‐RT using C‐ions for head and neck malignancies after photon RT is an effective treatment with tolerable toxicities. Further investigations, preferably in prospective trials, are required for greater reliability.

## CONFLICT OF INTEREST

None.

## AUTHOR CONTRIBUTION

DT was responsible for the methodology, analysis, and writing and editing of the manuscript. YD, MK, NK, and HS were responsible for clinical conceptualization and final editing of the manuscript. HI, TO, YS, TO, and HT were responsible for supervision.

## ETHICS STATEMENT

Ethics approval, patient consent, and clinical trial registration statements are provided in the Methods section.

## Supporting information


Appendix S1

## Data Availability

Research data are stored in an institutional repository. Secondary use of study data will be considered upon a request and approval of IRB.
