# Supplementary material for: Multicenter study of re‐irradiation using carbon‐ions for head and neck malignancies after photon radiotherapy
Source: Cancer Med. 2022 Apr 7;11(19):3593–601. doi: 10.1002/cam4.4741 (PMC9554451; doi:10.1002/cam4.4741)
Supplement: Supplementary file 1 — Appendix S1 [file CAM4-11-3593-s001.pdf]

Figure E1
Kaplan–Meier's curves of (a) local control, (b) progression-free survival and (c) overall survival classified into recurrent tumor and second primary tumor

(a)

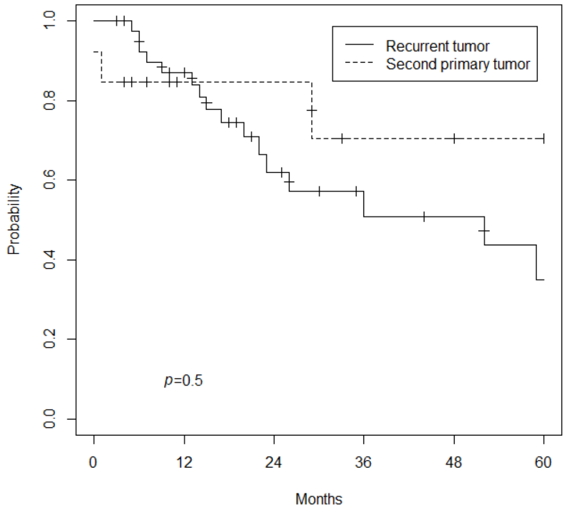

Number at risk

Recurrent tumor	43	31	14	9	7	4
Second primary tumor	13	6	6	3	3	1

(b)

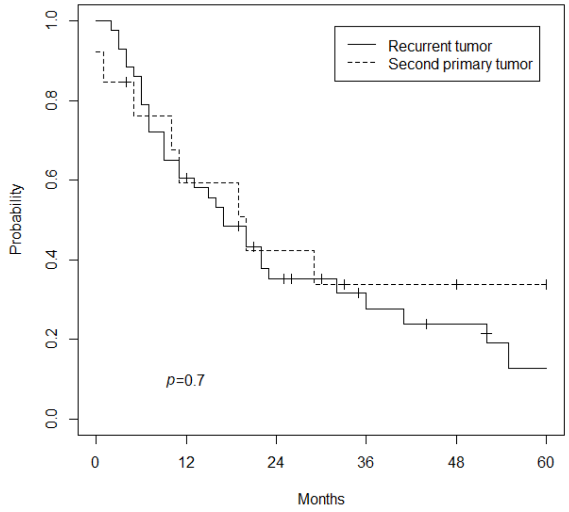

Number at risk

Recurrent tumor	43	26	13	8	5	2
Second primary tumor	13	7	5	3	3	1

(c)

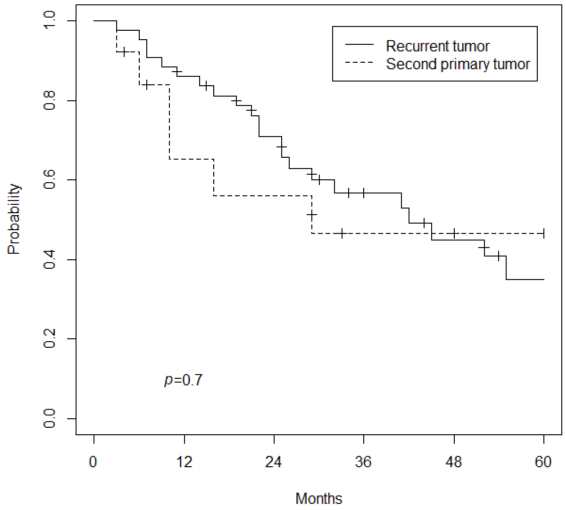

Number at risk

Recurrent tumor	43	36	27	16	11	6
Second primary tumor	13	7	6	3	3	1

Table E1
Late toxicities

Late toxicities (Grade ≥2)	Grade 2	Grade 3	Grade 4	Grade 5	Total
Dermatitis radiation	1			1	2
Optic nerve disorder	1		4		5
Oral hemorrhage			1		1
Glaucoma			1		1
Pharyngeal mucositis			1		1
Trismus	2	2			4
Dysphagia		2			2
Central nervous system disorder	2	1			3
Cataract		1			1
Cerebrospinal fluid leakage		1			1
Epistaxis		1			1
Pain		1			1
Oculomotor nerve disorder	2				2
Head soft tissue necrosis	1				1
Vestibular disorder	1				1
Abducens nerve disorder	1				1
Osteonecrosis of jaw	1				1
Middle ear inflamation	1				1
Recurrent laryngeal nerve palsy	1				1
Oral dysesthesia	1				1
Encephalitis infection	1				1
Nervous system disorder - Other, panhypopituitarism	1				1
Musculoskeletal and connective tissue disorder - Other, osteonecrosis	1				1
Total	18	9	7	1	35

Table E2
Late toxicities summarized by site of irradiation (Re-RT)

Site of irradiation (Re-RT)	(No. of patients)	Grade 2	Grade 3	Grade 4	Grade 5
Sinonasal cavities	(8/20)	5	3	2	1
Skull base	(6/12)	8	3	1	
Orbit	(3/7)		1	2	
Major salivary gland	(1/3)	1			
Acoustic organ	(1/3)	2			
Pharynx	(0/2)				
Oral cavity	(0/2)				
Others	(4/7)	2	2	2	
Total	(23/56)	18	9	7	1

Abbreviations: Re-RT, re-irradiation.

Table E3
Late toxicities summarized by PTV overlap

PTV overlap	(No. of patients)	Grade 2	Grade 3	Grade 4	Grade 5
Yes	(19/48)	14	7	5	1
No	(1/2)		1		
Unknown	(3/6)	4	1	2	

Abbreviations: PTV, Planning target volume.
